# Resveratrol directly targets DDX5 resulting in suppression of the mTORC1 pathway in prostate cancer

**DOI:** 10.1038/cddis.2016.114

**Published:** 2016-05-05

**Authors:** T Taniguchi, Y Iizumi, M Watanabe, M Masuda, M Morita, Y Aono, S Toriyama, M Oishi, W Goi, T Sakai

**Affiliations:** 1 Department of Molecular-Targeting Cancer Prevention, Graduate School of Medical Science, Kyoto Prefectural University of Medicine, Kawaramachi-Hirokoji, Kamigyo-ku, Kyoto 602-8566, Japan; 2 Department of Urology, Graduate School of Medical Science, Kyoto Prefectural University of Medicine, Kawaramachi-Hirokoji, Kamigyo-ku, Kyoto 602-8566, Japan

## Abstract

Resveratrol has various attractive bioactivities, such as prevention of cancer, neurodegenerative disorders, and obesity-related diseases. Therefore, identifying its direct binding proteins is expected to discover druggable targets. Sirtuin 1 and phosphodiesterases have so far been found as the direct molecular targets of resveratrol. We herein identified 11 novel resveratrol-binding proteins, including the DEAD (Asp-Glu-Ala-Asp) box helicase 5 (DDX5, also known as p68), using resveratrol-immobilized beads. Treatment with resveratrol induced degradation of DDX5 in prostate cancer cells. Depletion of DDX5 caused apoptosis by inhibiting mammalian target of rapamycin complex 1 (mTORC1) signaling. Moreover, knockdown of DDX5 attenuated the inhibitory activities of resveratrol against mTORC1 signaling and cancer cell growth. These data show that resveratrol directly targets DDX5 and induces cancer cell death by inhibiting the mTORC1 pathway.

Resveratrol, a dietary phytochemical enriched in wine, is attracting increasing attention because of its appealing bioactivities, such as prevention of cancer,^1, 2^ coronary heart disease,^3, 4^ neurodegenerative disorders,^5, 6^ and obesity-related diseases,^7, 8^ as well as extending lifespan.^9^ Because of these bioactive potentials, resveratrol has been tested in clinical trials and widely consumed as dietary supplements.^10, 11, 12^ To more clearly understand how resveratrol exerts these bioactivities, the direct target molecules of resveratrol have been investigated.^13^ Screening for the activators of sirtuin 1, which was previously considered necessary for the longevity achieved by caloric restriction,^14^ revealed that resveratrol directly activated sirtuin 1.^9^ However, several studies showed that resveratrol indirectly activated sirtuin 1.^15, 16^ Resveratrol was subsequently reported to activate sirtuin 1 by directly inhibiting phosphodiesterases (PDEs)^17^ and has recently been suggested again to directly activate sirtuin 1.^18^ Regardless of this controversy, these direct target molecules such as sirtuin 1 and PDEs cannot sufficiently account for other diverse molecular actions of resveratrol. In order to completely comprehend how resveratrol exerts its attractive bioactivities, it is necessary to fully uncover its direct target molecules and clarify the roles of these targets. Furthermore, identifying the direct targets of resveratrol is expected to lead to the discovery of druggable targets.^19^

Resveratrol modulates multiple signaling pathways, for example, by inhibiting the mammalian target of rapamycin complex 1 (mTORC1) pathway.^13, 20^ The mTORC1 pathway is known to be deregulated in various human diseases, such as malignant tumors, obesity, type II diabetes, and neurodegenerative diseases.^21^ Especially in malignancies, mTORC1 signaling promotes growth, survival, invasion, metastasis, and angiogenesis,^22, 23^ and mTORC1 inhibitors are used for cancer therapy.^21^ mTORC1 signaling is regulated by divergent pathways and molecules, such as the phosphatidylinositol 3-kinase pathway,^24^ mitogen-activated protein kinase pathway,^25^ AMP-activated protein kinase (AMPK) pathway,^26^ and astrin.^27^ However, the regulation of the mTORC1 pathway has yet to be clarified and elucidating this will contribute to the development of novel strategies to treat various diseases.

RNA-binding proteins are frequently deregulated in human diseases, such as cancer and neurodegenerative disorders.^28, 29^ DEAD (Asp-Glu-Ala-Asp) box helicase 5 (DDX5) is an RNA-binding protein that is overexpressed in various malignant tumors, such as prostate cancer, lung cancer, and ovarian cancer.^30^ The *DDX5* gene was shown to be amplified in breast cancer^31^ and fused with *ETV4*, the E26 transformation-specific transcription factor family gene, in prostate cancer.^32^ DDX5 promotes growth,^33^ metastasis,^34^ and drug resistance^35^ by activating several oncogenic pathways.^34, 36^ Although DDX5 also functions as a transcriptional co-activator of the androgen receptor in hormone-dependent prostate cancer,^37^ its roles in hormone-independent prostate cancer have not been clarified.

The present study shows that the inhibition of the mTORC1 pathway and cancer growth by resveratrol is not attributed to the inhibition of PDE. To identify the novel targets of resveratrol, we produced resveratrol-immobilized beads using our previously reported method.^38^ The affinity beads method and proteomic analysis identified 11 novel targets, including DDX5. Resveratrol directly bound to and promoted metalloprotease-dependent degradation of DDX5 protein, leading to cancer cell death with inhibition of the mTORC1 pathway. We demonstrate for the first time that DDX5 is a primary target of resveratrol in tumor suppression.

## Results

### Resveratrol, but not the PDE4 inhibitor rolipram, inhibits the mTORC1 pathway and growth of prostate cancer cells

**Figure 1:**
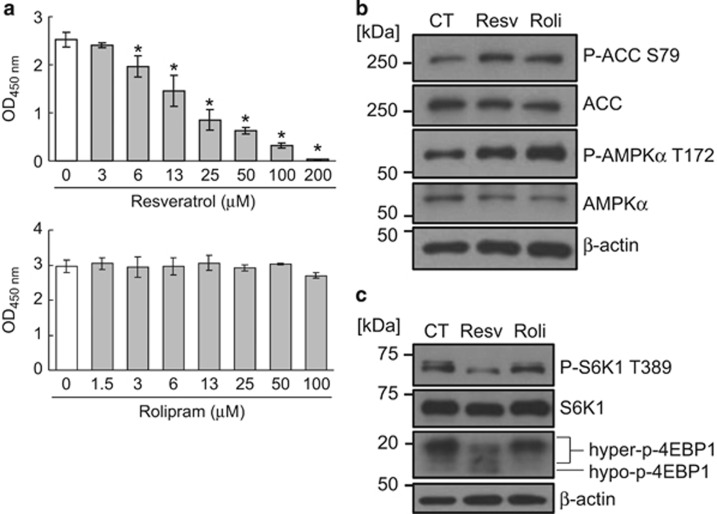
Resveratrol, but not a PDE inhibitor, suppresses the growth of prostate cancer cells. (**a**) Human prostate cancer PC-3 cells were treated with the indicated concentrations of resveratrol or the PDE4 inhibitor rolipram for 72 h. Relative viability of the cells was measured by CCK-8 assay. Data are means±S.D. (*n*=3). *P<0.05 relative to control (one-way analysis of variance, Bonferroni *post-hoc* tests). (**b** and **c**) Western blotting analysis of PC-3 cells treated with 0.1% DMSO (CT), 100 *μ*M resveratrol (Resv), or 100 *μ*M rolipram (Roli) for 3 h (**b**) or 24 h (**c**)

Resveratrol activates the AMPK pathway and restores aging-related metabolic phenotypes by directly inhibiting PDEs.^17^ To evaluate the roles of PDEs in cancer growth inhibition by resveratrol, we compared the effects of resveratrol with those of the specific PDE4 inhibitor rolipram on the growth of prostate cancer PC-3 cells. As shown in Figure 1a, resveratrol, but not rolipram, inhibited the growth of PC-3 cells. It was reported that AMPK inhibited the mTORC1 pathway,^26^ which promotes the development of prostate cancer,^23^ and also that resveratrol inhibited the mTORC1 pathway.^7, 39^ We therefore examined whether these PDE inhibitors, including resveratrol, inhibited the mTORC1 pathway. Although inhibition of PDE increased the phosphorylation of AMPK*α* at Thr172 and its substrate acetyl-CoA carboxylase (ACC) at Ser79 (Figure 1b), indicating the activation of AMPK, only resveratrol inhibited the phosphorylation of ribosomal protein S6 kinase 1 (S6K1) at Thr389 and eukaryotic translation initiation factor 4E-binding protein 1 (4EBP1), reflecting the activation of mTORC1 (Figure 1c). These results suggest that resveratrol suppresses the mTORC1 pathway and growth of prostate cancer cells independent of the inhibition of PDE.

### Identification of DDX5 as a target of resveratrol

**Figure 2:**
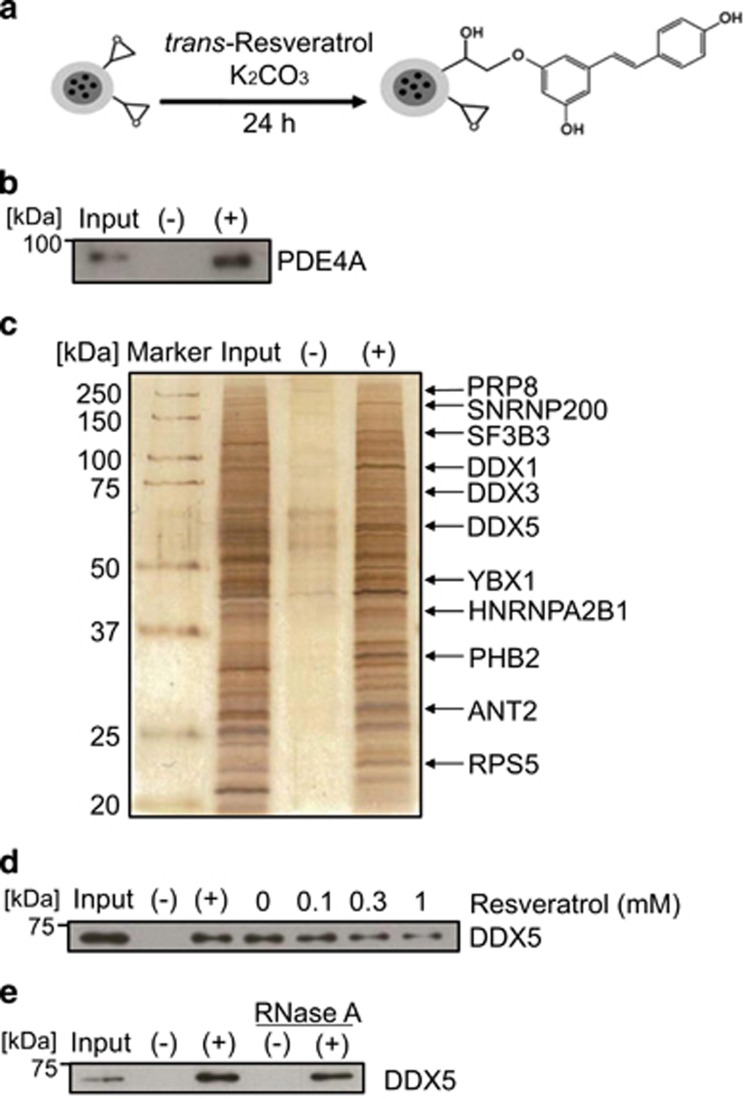
Identification of resveratrol-binding proteins. (**a**) The scheme for fixation of resveratrol onto magnetic FG beads with epoxy linkers. (**b**) Purified recombinant PDE4A (2 *μ*g) was incubated with empty (−) or resveratrol-immobilized (+) beads for 4 h, and bound PDE4A was detected by western blotting. The input lane corresponds to recombinant PDE4A (250 ng). (**c**) Resveratrol-binding proteins were purified from human prostate cancer PC-3 cell extracts, silver-stained, and identified by matrix assisted laser desorption/ionization time-of-flight mass spectrometric analysis. The input lane represents 1% of the PC-3 cell extracts used for the binding assay. (**d**) In the competitive assay, PC-3 cell extracts were preincubated with the indicated doses of resveratrol for 1 h and incubated with the beads for 15 min. Bound DDX5 was detected by western blotting. The input lane represents 5% of the PC-3 cell extracts used for the binding assay. (**e**) Purified recombinant DDX5 (1 *μ*g) with or without RNase A was incubated with the beads, and bound DDX5 was detected by western blotting. The input lane corresponds to recombinant DDX5 protein (150 ng)

As the inhibition of PDE could not suppress the mTORC1 pathway and cancer growth, we speculated the existence of unknown target molecules of resveratrol that were related to its anticancer activity. To identify the novel molecular targets of resveratrol, we generated resveratrol-immobilized beads using our previously described method^38^ (Figure 2a). We first confirmed that purified recombinant PDE4A protein bound to resveratrol-fixed beads (Figure 2b). Resveratrol-binding proteins were then purified from PC-3 whole-cell extracts using these beads and analyzed by MALDI-TOF MS. The resveratrol-fixed beads method and proteomic analysis uncovered 11 novel resveratrol-binding proteins, including 7 RNA-binding proteins, 2 mitochondrial proteins, 1 transcription factor, and 1 ribosomal protein (Figure 2c and Supplementary Table S1). As one of the resveratrol-binding proteins, the RNA helicase DDX5/p68 was reported to be overexpressed and amplified in several malignant tumors and have oncogenic properties,^30, 31^ we focused on the role of DDX5 as a target of resveratrol in cancer inhibition. The competitive assay showed that the binding of DDX5 to resveratrol-fixed beads was competed with free resveratrol, suggesting that resveratrol specifically bound to DDX5 (Figure 2d). Furthermore, purified recombinant DDX5 bound to resveratrol-fixed beads regardless of the digestion of RNA by RNase A treatment (Figure 2e). These findings indicate that resveratrol directly binds to DDX5.

**Figure 3:**
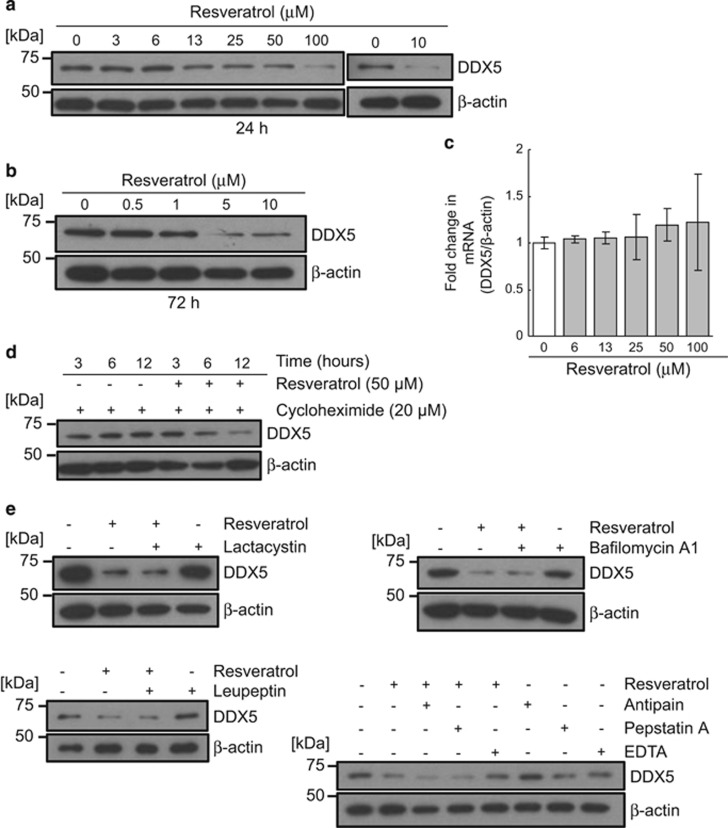
Treatment with resveratrol degrades DDX5 protein. (**a** and **b**) PC-3 cells were treated with the indicated concentrations of resveratrol for 24 h (**a**) or every 24 h for 72 h (**b**). DDX5 protein was detected by western blotting. (**c**) Quantitative reverse transcriptase–PCR analysis of DDX5 mRNA in PC-3 cells treated with resveratrol for 24 h. Data are means±S.D. (*n*=3). (**d**) PC-3 cells were incubated for the indicated times with or without 50 *μ*M resveratrol and/or 20 *μ*M cycloheximide. DDX5 protein was detected by western blotting. (**e**) PC-3 cells were incubated for 24 h with or without 20 *μ*M lactacystin, 1 *μ*M bafilomycin A1, 100 *μ*M leupeptin, 100 *μ*M antipain, 1 *μ*M pepstatin A, or 10 mM EDTA and/or 50 *μ*M resveratrol. DDX5 protein was detected by western blotting

We next evaluated the effects of resveratrol on endogenous DDX5. The endogenous protein levels of DDX5 were dose-dependently decreased by the treatment with resveratrol in PC-3 and DU145 cells (Figure 3a and Supplementary Figure S1a). We further investigated whether the physiological concentrations of resveratrol also downregulated DDX5. The once-daily administration of 5 g of resveratrol to healthy volunteers for 29 days was shown to yield 2.3–7.0 *μ*M of resveratrol in the plasma.^40^ The protein levels of DDX5 were reduced (Figure 3b) after treatment with clinically achievable concentrations of resveratrol (5–10 *μ*M) every 24 h for 3 days. These data raise the possibility that DDX5 is a physiological target of resveratrol in the human body.

We further examined the mechanism by which resveratrol downregulated the expression of DDX5 protein. Treatment with resveratrol did not downregulate DDX5 mRNA (Figure 3c). As shown in Figure 3d, DDX5 protein was stable because it was not decreased by the *de novo* protein synthesis inhibitor cycloheximide, but resveratrol reduced DDX5 protein in the presence of cycloheximide, indicating that resveratrol promoted the degradation of DDX5 protein. Next we tested what types of proteases were related to degradation of DDX5 protein. Only EDTA partially inhibited the degradation of DDX5 by resveratrol, whereas the proteasome inhibitor lactacystin, the autophagy inhibitor bafilomycin A1, and protease inhibitors (leupeptin, antipain, and pepstatin A) did not (Figure 3e). These results suggest that resveratrol degrades DDX5 protein by promoting metalloprotease-dependent degradation.

### Depletion of DDX5 expression suppresses the growth of prostate cancer cells by inhibiting the mTORC1 pathway and inducing apoptosis

**Figure 4:**
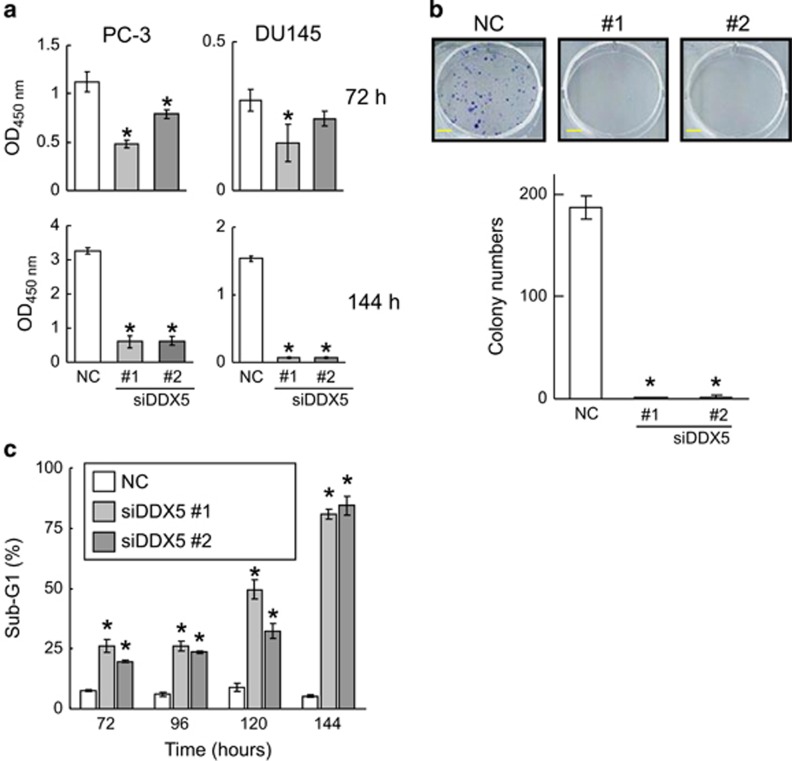
Knockdown of DDX5 inhibits the growth of hormone-independent prostate cancer cells. (**a**) PC-3 and DU145 cells were transfected with a negative control siRNA (NC), siDDX5 #1, or siDDX5 #2 for 72 or 144 h. Relative viability of the cells was measured by CCK-8 assay. Data are means±S.D. (*n*=3). *P<0.05 relative to control (one-way analysis of variance (ANOVA), Bonferroni *post-hoc* tests). (**b**) After PC-3 cells were transfected with or without siDDX5 and incubated for 9 days, colonies were stained with crystal violet and counted (scale bar: 5 mm). Data are means±S.D. (*n*=3). *P<0.05 relative to control (one-way ANOVA, Bonferroni *post-hoc* tests). (**c**) PC-3 cells were transfected with or without siDDX5 and incubated for 72, 96, 120, or 144 h, and the percentage of sub-G1 population was quantified by flow cytometry. Data are means±S.D. (*n*=3). *P<0.05 relative to control (one-way ANOVA, Bonferroni *post-hoc* tests)

Although DDX5 is overexpressed in prostate cancer and functions as a co-activator of the androgen receptor,^37^ its functions in hormone-refractory prostate cancer remain unknown. We found that knockdown of DDX5 inhibited the growth and colony formation of hormone-refractory prostate cancer PC-3 and DU145 cells (Figures 4a and b), similar to the treatment with resveratrol (Figure 1a and Supplementary Figure S1b). Knockdown of DDX5 remarkably induced apoptosis in PC-3 cells (Figure 4c), similar to the resveratrol treatment (Supplementary Figure S2). These results suggest that depletion of DDX5 inhibits the growth of hormone-refractory prostate cancer cells with inducing apoptosis.

**Figure 5:**
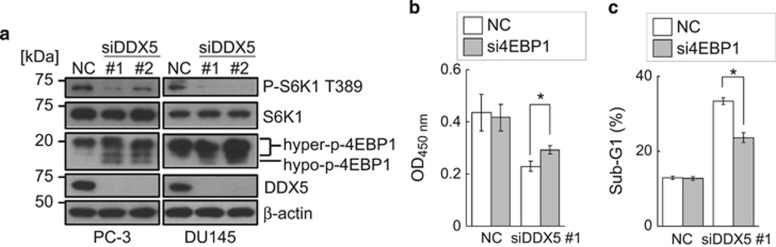
Knockdown of DDX5 inhibits the mTORC1 pathway of hormone-independent prostate cancer cells. (**a**) Western blotting analysis was performed after PC-3 and DU145 cells were transfected with or without siDDX5 and incubated for 72 h. (**b** and **c**) After PC-3 cells were transfected with or without siDDX5 #1 and/or si4EBP1 for 72 h, CCK-8 assay (**b**) and quantification of apoptosis (**c**) were performed. Data are means±S.D. (*n*=3). *P<0.05 relative to control (Student's *t*-test)

We then investigated how depletion of DDX5 inhibited the growth of prostate cancer cells. Although depletion of DDX5 was reported to induce apoptosis by downregulating Notch1 signaling in lymphoblastic leukemia cells,^36^ Notch1 signaling was not suppressed by DDX5 depletion in prostate cancer cells (Supplementary Figure S3). We next examined whether knockdown of DDX5 inhibited mTORC1 signaling similar to the treatment with resveratrol (Figure 1c and Supplementary Figure S1a). As shown in Figure 5a, DDX5 knockdown inhibited the mTORC1 pathway in prostate cancer PC-3 and DU145 cells. As dephosphorylation of 4EBPs is critical for causing growth inhibition and apoptosis,^41^ we tested whether knockdown of only 4EBP1, one of the 4EBP family, attenuated the inhibitory effects of DDX5 depletion. The knockdown of 4EBP1 partially inhibited the growth inhibition and apoptosis induced by the depletion of DDX5 (Figures 5b and c and Supplementary Figure S4). These findings indicate that depletion of DDX5 can mimic the inhibitory effects of resveratrol against the mTORC1 pathway and prostate cancer cell growth.

### Resveratrol inhibits the mTORC1 pathway and growth of prostate cancer cells by targeting DDX5

**Figure 6:**
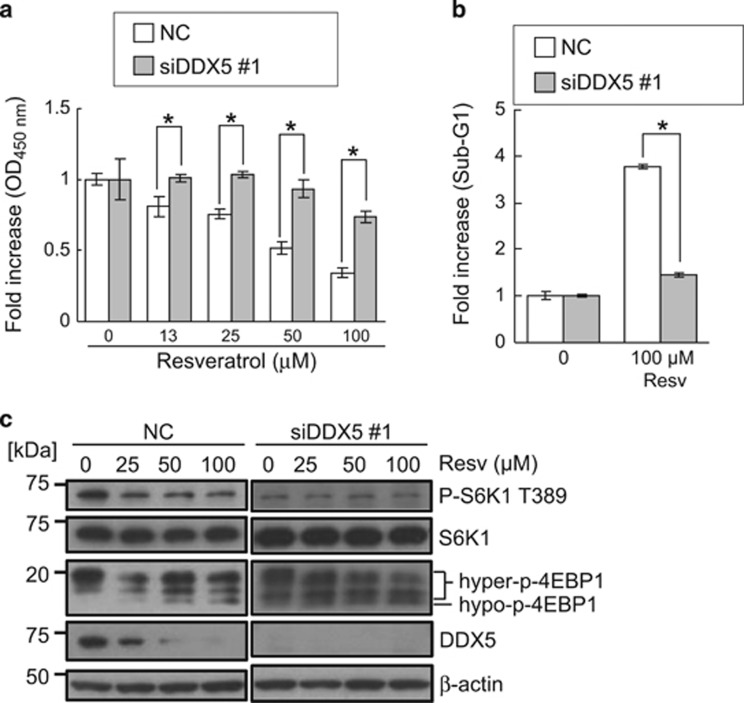
Resveratrol inhibits the mTORC1 pathway and growth of prostate cancer cells through depletion of DDX5. PC-3 cells were treated with resveratrol at the indicated concentrations for 24 h after transfected with a negative control siRNA (NC) or siDDX5 #1 and incubated for 48 h. (**a**) Relative viability was measured by CCK-8 assay. Data are means±S.D. (*n*=3). *P<0.05 relative to control (Student's *t*-test). (**b**) Apoptosis was quantified by flow cytometric analysis. Data are means±S.D. (*n*=3). *P<0.05 relative to control (Student's *t*-test). Resv; Resveratrol. (**c**) mTORC1 activity was examined by western blotting

**Figure 7:**
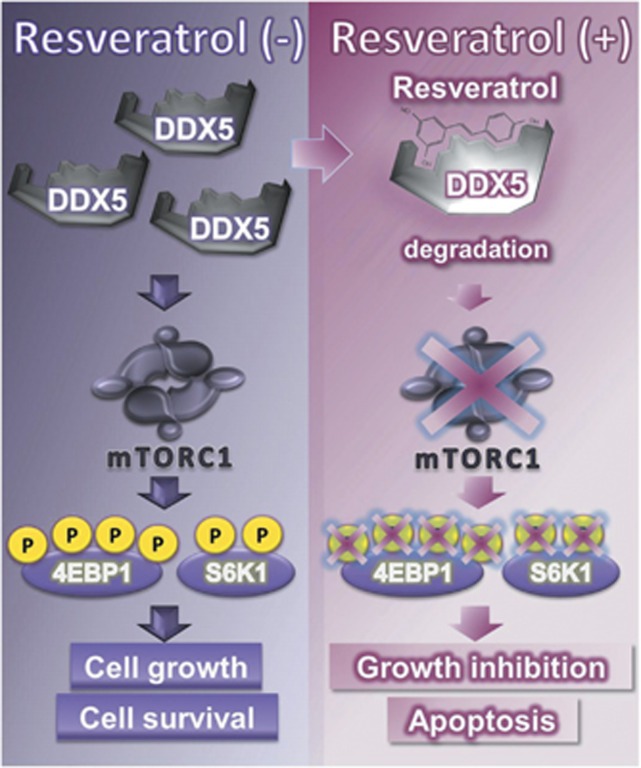
A schematic illustration of targeting the DDX5–mTORC1 axis by resveratrol. DDX5 protein is overexpressed in castration-resistant prostate cancer and promotes cell survival and growth by activating mTORC1 signaling. Resveratrol directly binds to DDX5 protein and promotes degradation of DDX5 protein, leading to suppression of the mTORC1 pathway, cancer cell survival, and growth

As resveratrol degraded DDX5 protein and the depletion of DDX5 inhibited the mTORC1 pathway and growth of prostate cancer cells, we evaluated the significance of degrading DDX5 in the anticancer effects of resveratrol. As shown in Figures 6a and b, the depletion of DDX5 attenuated the growth inhibition and apoptosis caused by resveratrol. Furthermore, the knockdown of DDX5 also attenuated the inhibitory effects of resveratrol on the mTORC1 pathway (Figure 6c). These results suggest that resveratrol inhibits the growth of prostate cancer cells by directly targeting DDX5 (Figure 7).

## Discussion

In the present study, we employed resveratrol-immobilized beads to explore the unknown targets of resveratrol and identified 11 resveratrol-binding proteins. One of the resveratrol-binding proteins, DDX5, was degraded by treatment with physiological concentrations of resveratrol. The depletion of DDX5 protein resulted in growth inhibition and cell death by suppressing mTORC1 signaling in androgen-independent prostate cancer cells, similar to the treatment with resveratrol. Furthermore, these activities of resveratrol were repressed by DDX5 depletion. Taken together, our results suggest that resveratrol suppresses mTORC1 signaling in androgen-independent prostate cancer cells by directly targeting DDX5 (Figure 7), and 10 other resveratrol-binding proteins might also be druggable targets, similar to DDX5. As shown in Supplementary Figure S5, we also found that resveratrol at 100 *μ*M, but not 50 *μ*M, downregulated mRNA expressions of transglutaminase-4 (TGM4) and prostate leucine zipper (PrLZ). As TGM4 promotes cellular migration^42^ and PrLZ suppresses apoptosis,^43^ a high concentration of resveratrol might further exhibit other anticancer effects by targeting other resveratrol-binding proteins and downregulating these molecules.

As resveratrol exerts beneficial bioactivities in organisms, its target proteins have been investigated. Although sirtuin 1, one of the most famous targets of resveratrol, was activated by 25 *μ*M of resveratrol in cells,^18^ it was difficult for resveratrol to reach 25 *μ*M under physiological conditions.^40^ In contrast, it was suggested that resveratrol at 10 *μ*M activated the AMPK pathway by directly inhibiting PDEs in cells.^17^ Our study showed that resveratrol degraded DDX5 protein at 5 *μ*M every 24 h for 3 days (Figure 3b). Taken together, DDX5 as well as PDEs may be targeted by resveratrol in organisms, including human beings.

There are currently few efficient agents for castration-resistant prostate cancer.^44^ In this study, we have suggested that resveratrol inhibits the growth of castration-resistant prostate cancer cells by degrading DDX5. DDX5 is overexpressed and promotes tumor development in prostate and other malignant tumors.^30^ These data raise the possibility that DDX5 is a druggable target for castration-resistant prostate cancer therapy. On the other hand, by screening for p15-inducing compounds, we have discovered the MEK inhibitor trametinib,^45^ which has recently been approved for the treatment of BRAF-mutated melanoma by the US Food and Drug Administration, EU, and others. Screening for DDX5-targeting agents may provide effective drugs for castration-resistant prostate cancer.

At present, docetaxel and antiandrogen agents are mainly utilized in the treatment of castration-resistant prostate cancer. However, as these agents are not sufficient, effective combination therapies are required. As resveratrol and the antiandrogen flutamide synergistically inhibit the androgen receptor,^46^ the combination of resveratrol and flutamide may be a promising therapy. Moreover, it has been reported that the mTORC1 inhibitor rapamycin enhances docetaxel efficacy against the PC-3 xenograft model, a castration-resistant prostate cancer model, by downregulating survivin.^47^ In the present study, resveratrol inhibited the mTORC1 pathway by targeting DDX5, and we raise the possibility that resveratrol may augment docetaxel efficacy against castration-resistant prostate cancer.

The present study suggested that resveratrol inhibits the mTORC1 pathway by targeting DDX5. Previous studies indicated that the suppression of mTORC1 signaling led not only to the inhibition of cancer progression^22^ but also to the amelioration of neurodegenerative disorders,^48^ alleviation of obesity-related diseases,^49^ and extension of lifespans.^50^ Therefore, resveratrol may exert these clinical benefits^6, 9, 12^ by targeting DDX5.

## Materials and Methods

### Reagents

*trans*-Resveratrol (Cat. No. 70675) and rolipram (Cat. No. 10011132) were purchased from Cayman Chemical (Ann Arbor, MI, USA). Purified recombinant proteins of *Homo sapiens* DEAD box helicase 5 (TP300371) and *Homo sapiens* phosphodiesterase 4A, cAMP-specific (TP307765) were purchased from OriGene Technologies (Rockville, MD, USA). Cycloheximide (Cat. No. 06741-91) and pepstatin A (Cat. No. 26436-52) were purchased from Nacalai Tesque (Kyoto, Japan). Bafilomycin A1 (Cat. No. B-1080) was purchased from LC Laboratories (Woburn, MA, USA). Lactacystin (Cat. No. 4368-v), leupeptin (Cat. No. 4041), and antipain (Cat. No. 4062) were purchased from Peptide Institute (Osaka, Japan).

### Cell culture

The human prostate cancer cell lines PC-3 and DU145 were obtained as the cell lines of NCI-60 from the NCI Developmental Therapeutics Program. These cells were cultured in RPMI 1640 medium supplemented with 10% fetal bovine serum, 2 mM l-glutamine, 50 units/ml penicillin, and 100 *μ*g/ml streptomycin at 37 °C in 5% CO_2_.

### Cell viability assay

Cell viability was determined as previously described.^38^ Briefly, after the Cell Counting Kit-8 (CCK-8) solution (Dojindo Laboratories, Kumamoto, Japan) was added to the medium and incubated, the absorbance (450 nm) of the samples was measured.

### Western blotting analysis

Western blotting analysis was performed as previously described.^38^ For detection of AMPK activation, PC-3 cells were fixed with ice-cold 10% trichloroacetic acid in PBS on ice for 30 min. After scraping and washing with PBS, the cells were lysed with RIPA buffer and sonicated. The supernatants were subjected to SDS-PAGE and analyzed by western blotting. Anti-DDX5 (PAb204, 05-850, Millipore, Billerica, MA, USA), 4EBP1 (#9644, Cell Signaling Technology, Danvers, MA, USA), P-S6K T389 (#9234, Cell Signaling Technology), S6K (#2708, Cell Signaling Technology), P-ACC S79 (#3661, Cell Signaling Technology), ACC (#3676, Cell Signaling Technology), P-AMPK*α* T172 (#2535, Cell Signaling Technology), AMPK*α* (#2603, Cell Signaling Technology), PDE4 (ab14628, Abcam, Cambridge, UK), and *β*-actin (A5441, Sigma-Aldrich, St. Louis, MO, USA) antibodies were used.

### Preparation of resveratrol-immobilized beads

Magnetic FG beads with epoxy linkers were purchased from Tamagawa Seiki (Nagano, Japan). The fixation of resveratrol onto the beads was performed as previously described.^38^ Briefly, the beads were incubated with 10 mM *trans*-resveratrol in *N, N*-dimethylformamide (DMF) containing potassium carbonate at 37 °C for 24 h. After being washed twice with DMF, the beads were then washed with Milli-Q water.

### Purification and identification of resveratrol-binding proteins

Purification and identification of resveratrol-binding proteins were performed using resveratrol-fixed beads as previously described.^38^ In the competitive binding assay, cell extracts were incubated with free *trans*-resveratrol for 1 h before incubation with the beads for 15 min. Recombinant DDX5 protein was preincubated with or without 150 *μ*g/ml RNase A (R4642, Sigma-Aldrich) for 30 min at room temperature and used for the binding assay.

### Quantitative real-time RT-PCR

Quantitative RT-PCR analysis was performed as previously described.^51^ Complementary DNA was amplified and quantified using TaqMan probes (Applied Biosystems, Foster City, CA, USA) to DDX5 (Hs01075383_g1), HES1 (Hs00172878_m1), kallikrein 4 (KLK4) (Hs00191772_m1), prostate carcinoma tumor antigen 1 (PCTA1) (Hs01057135_m1), TGM4 (Hs00162710_m1), PrLZ (Hs00893105_m1), and *β*-actin (Cat. No. 4352935E).

### RNAi

The following siRNAs (Life Technologies, Carlsbad, CA, USA) were used: siDDX5 #1 (HSS102717), 5′-GGUGCAGCAAGUAGCUGCUGAAUAU-3′ siDDX5 #2 (HSS102715), 5′-GGAAUCUUGAUGAGCUGCCUAAAUU-3′ si4EBP1 (HSS141934), 5′-GGGUCACCAGCCCUUCCAGUGAUGA-3′ and a negative control siRNA (Cat. No. 12935-115). Only sense strands are shown. Transfection of siRNAs was performed as previously described.^38^

### Colony-formation assay

PC-3 cells were seeded at a density of 200 cells/well in six-well plates. The cells were transfected with each siRNA and incubated for 9 days. After being fixed with 10% formalin, the colonies were stained with crystal violet. The total number of colonies was counted.

### Quantification of apoptosis

Apoptosis was detected and quantified as previously described.^51^ Briefly, after washing with PBS, PC-3 cells were suspended in PBS containing 0.1% Triton X-100, 50 *μ*g/ml propidium iodide, and 100 *μ*g/ml RNase A. The percentage of hypodiploid DNA (sub-G1) was quantified as the ratio of apoptosis by FACSCalibur (Becton, Dickinson and Company, Franklin Lakes, NJ, USA).

### Statistics

Data are represented as means and S.D. Statistical analyses were performed using one-way ANOVA followed by Bonferroni *post-hoc* tests or unpaired Student's *t*-test.

## Abbreviations

DDX5: DEAD (Asp-Glu-Ala-Asp) box helicase 5
mTORC1: mammalian target of rapamycin complex 1
PDE: phosphodiesterase
AMPK: AMP-activated protein kinase
ACC: acetyl-CoA carboxylase
4EBP1: eukaryotic translation initiation factor 4E-binding protein 1
DMF: *N, N*-dimethylformamide
TGM4: transglutaminase-4
PrLZ: prostate leucine zipper
