# Supplementary material for: Resveratrol directly targets DDX5 resulting in suppression of the mTORC1 pathway in prostate cancer
Source: Cell Death Dis. 2016 May 5;7(5):e2211–. doi: 10.1038/cddis.2016.114 (PMC4917653; doi:10.1038/cddis.2016.114)
Supplement: Supplementary Information [file cddis2016114x1.doc]

**Supplementary Figure 1** Resveratrol inhibits the mTORC1 pathway and growth of prostate cancer cells. (**a**) Western blot analysis was performed after treatment of PC-3 and DU145 cells with resveratrol at the indicated concentrations for 24 h. (**b**) DU145 cells were treated with resveratrol at the indicated concentrations for 72 h. Relative viability was measured by CCK-8 assay. Data are means ± S.D. (*n* = 3). **P* < 0.05 relative to control (One-way ANOVA, Bonferroni *post-hoc* tests)

**Supplementary Figure 2** Resveratrol induces apoptosis in prostate cancer PC-3 cells. PC-3 cells were treated with the indicated concentrations of resveratrol for 24 or 144 h, and the percentage of the sub-G1 apoptotic population was quantified as the ratio of apoptosis. Data are means ± S.D. (*n* = 3). **P* < 0.05 relative to control (One-way ANOVA, Bonferroni *post-hoc* tests)

**Supplementary Figure 3** Knockdown of DDX5 does not inhibit Notch1 signaling in PC-3 cells. qRT-PCR analysis of HES1 and β-actin mRNA was performed after PC-3 cells were transfected with a negative control siRNA (NC), siDDX5 #1, or siDDX5 #2 and incubated for 72 h. Data are means ± S.D. (*n* = 3)

**Supplementary Figure 4** Western blot analysis after knockdown of DDX5 and/or 4EBP1 in PC-3 cells. After PC-3 cells were transfected with or without siDDX5 #1 and/or si4EBP1 for 72 h, Western blot analysis was performed

**Supplementary Figure 5** Effects of resveratrol on mRNA expressions of prostate associated markers in PC-3 cells. After PC-3 cells were treated with the indicated doses of resveratrol for 24 h, mRNA expressions of four prostate associated markers were quantified by qRT-PCR analysis. Data are means ± S.D. (*n* = 3). **P* < 0.05 relative to control (One-way ANOVA, Bonferroni *post-hoc* tests)

**Supplementary Table 1** List of the resveratrol-binding proteins identified
